# Dominant Components of the Giant Panda Seminal Plasma Metabolome, Characterized by ^1^H-NMR Spectroscopy

**DOI:** 10.3390/ani12121536

**Published:** 2022-06-14

**Authors:** Chenglin Zhu, Lei Jin, Bo Luo, Qiang Zhou, Li Dong, Xiaoyan Li, Hemin Zhang, Yan Huang, Caiwu Li, Likou Zou, Luca Laghi

**Affiliations:** 1 College of Food Science and Technology, Southwest Minzu University, Chengdu 610041, China; chenglin.zhu@swun.edu.cn; 2 College of Resources, Sichuan Agricultural University, Chengdu 611130, China; jinleileileilei@outlook.com; 3 Key Laboratory of State Forestry and Grassland Administration on Conservation Biology of Rare Animals in the Giant Panda National Park, The China Conservation and Research Center for the Giant Panda, Dujiangyan 611800, China; boluo911@126.com (B.L.); zqzq13881725851@sina.com (Q.Z.); 13980177209@139.com (L.D.); lxy15808153955@163.com (X.L.); wolong_zhm@126.com (H.Z.); pandayard@hotmail.com (Y.H.); 4 Department of Agricultural and Food Sciences, University of Bologna, 47521 Cesena, Italy; l.laghi@unibo.it

**Keywords:** giant panda, seminal plasma, age, estrus, metabolomics, ^1^H-NMR

## Abstract

**Simple Summary:**

As China’s flagship animal, the giant panda (*Ailuropoda melanoleuca*) attracts much attention due to its small population and low natural reproductive rate. Therefore, artificial insemination has become the leading practical approach in the captive breeding programs of giant pandas worldwide. Seminal plasma acts as a medium between spermatozoa and the external stimuli, and its characteristics have been directly linked to fertility in both artificial insemination and natural fertilization. The current work, for the first time, attempts to characterize, by proton magnetic resonance spectroscopy (^1^H-NMR), the metabolome of healthy giant panda seminal plasma. A total of 35 molecules were quantified, with distinct age-related trends highlighted by a multivariate analysis, and the concentrations of 2,3-butanediol were significantly different between individuals younger than 8 years and older than 13 years. In addition, isopropanol’s concentration was significantly linked to estrus stages. Besides, the variations in the metabolome’s profile with storage time were also evaluated. This study may serve as a reference for research wishing to shed light on the biological mechanisms affecting giant panda sperm’s overall quality and may ultimately lead to novel approaches to giant panda artificial insemination.

**Abstract:**

As an assisted breeding technique, artificial insemination has become the main effective practical approach in the captive breeding programs of giant panda worldwide. The composition of seminal plasma plays an important role in the success of breeding. The present work is the first attempt to characterize, by proton magnetic resonance spectroscopy (^1^H-NMR), the metabolome of healthy giant panda seminal plasma. A total of 35 molecules were quantified, with the concentration of 2,3-butanediol being significantly different between individuals younger than 8 years and older than 13 years, and other distinct age-related trends were highlighted by a multivariate analysis. Isopropanol’s concentration was significantly linked to estrus stages. Besides, the variations in the metabolome’s profile during storage were also evaluated. This study may serve as a reference for further research wishing to shed light on the biological mechanisms affecting giant panda sperm’s overall quality and may ultimately lead to novel approaches to giant panda artificial insemination.

## 1. Introduction

As the flagship animal of China, the giant panda (*Ailuropoda melanoleuca*) is one of the most endangered mammals on earth, which is attributed to its small population and limited reproductive rate [1]. Female giant pandas are in estrus for only a few days each year, so less than 10 percent of giant panda males mate, and less than 30 percent of females conceive naturally [2,3]. Therefore, artificial insemination has become the main practical approach in the captive breeding programs of giant pandas worldwide since it was first attempted in the 1970s [4,5,6].

To maximize the success rate of the procedure, the spermatozoa number, motility, and morphology have been widely evaluated [2,7,8]. As seminal plasma acts as a medium between spermatozoa and the external stimuli, its characteristics have also been directly linked to fertility [9]. Recent advances in high-throughput techniques (GC-MS (gas chromatography-mass spectrometry), LC-MS (liquid chromatograph-mass spectrometry), and ^1^H-NMR (proton magnetic resonance spectroscopy)) have shed light on the role played by its metabolome, the ensemble of its low-weight molecules. Metabolomics, the study of the metabolome, has therefore become a valuable tool for studying humans’ and other mammals’ seminal plasma [10,11,12]. For instance, Jayaraman et al. successfully distinguished different forms of male infertility through an NMR-based metabolomic approach and identified lysine as a potential biomarker for the detection and diagnosis of idiopathic infertility [12]. Mumcu et al. found potential biomarkers of infertility by studying the metabolome of seminal plasma in patients with idiopathic oligoasthenoteratozoospermia [13].

In humans and in any other animal, the age of the donor has been reported to be a key factor for seminal plasma perturbations, so the relationships between seminal plasma characteristics and age have been widely investigated. For instance, Fosse et al. found that older men exhibit a high pro-inflammatory seminal plasma profile and determine unexplained recurrent pregnancy losses [14]. Vince et al., studying bulls during cold and warm periods of the year, found age-related differences in the antioxidant profiles of their seminal plasma [15]. Such findings agree with those of Sicy et al., who assessed the seminal plasma oxidant/antioxidant status of Arab stallions of different ages [16]. In rams, Souza et al. found that the total protein concentration in the seminal plasma significantly increased during the first 28 weeks of life [17]. In Arabian horses, Ahmed et al. noticed significant age-related effects on seminal plasma’s biochemical constituents, namely, glycerylphosphorylcholine, ergothioneine, and total protein concentrations [18].

Interestingly, the estrus stages of the female giant panda have been found to affect male giant panda semen quality, both directly and indirectly. Tsutsui et al. found that male giant panda semen volume, sperm count, and testis volume markedly differed from 90 days before estrus until 66 days after estrus when a female giant panda was housed nearby. From this observation, they inferred that spermatogenesis was active in this male panda from approximately 3 months before estrus to 2 months after estrus in the adjacent female [3]. In addition, Palmer et al. reported that testes morphometry, fecal androgen excretion, seminal quality, and the panda’s overall behavior clearly demonstrated reproductive seasonality in the male giant panda [19].

Cryopreservation is considered to be the main form of sperm preservation and is widely used for artificial insemination. It is known that plasma characteristics may be altered during storage with consequences on spermatozoa motility [20]. However, most of the works on the topic have focused on the effects of dilution or removal, the most common practice prior to cryopreservation in most mammalian species [21]. Lower attention has been paid to the detailed study of the effects of storage time on the metabolome of seminal plasma.

The above works suggest that advances in the procedures for the artificial insemination of giant pandas may come from the study of the seminal plasma metabolome, which is the object of the present work. With the purpose of providing reliable quantitative information, an untargeted approach based on ^1^H-NMR was used by taking advantage of the high reproducibility of this analytical platform [22]. As the samples we were able to collect had been frozen and donated from animals covering all life stages of the giant panda, we had the opportunity to obtain preliminary information on how age, the estrus stages of the female giant panda, and storage time could influence the molecular profile of male giant panda seminal plasma. These findings may serve as a starting point for further research shedding light on the biological mechanisms impacting giant panda sperm’s overall quality and ultimately lead to novel approaches to giant panda artificial insemination.

## 2. Materials and Methods

### 2.1. Sample Collection

All samples were kindly provided, regardless of the purpose of this study, during the procedures for artificial insemination and cryogenic storage that are routinely conducted at the China Conservation and Research Center for the Giant Panda by selecting for metabolomics investigation an aliquot of sperm stored for insemination. Sperm freezing medium (TEST-yolk buffer with glycerol and gentamicin) and sperm refrigeration medium (TEST-yolk buffer with gentamicin) were added immediately after sample collection. Semen collection was achieved with the panda in dorsal recumbency. Prior to electroejaculation, feces were manually removed from the rectum, and the penis and surrounding area were washed with warm water. A 4.2 cm diameter, 20 cm long probe was used with three l cm circular electrodes. The probe was placed in the rectum to a depth of 15–20 cm. Electrostimulations were performed with a 110 V, 60 Hz machine with a 220 V, 50 Hz transformer. A range of 5–12 stimulations with 2–8 V (a maximum of 200 mA) over a 3–5 s period was repeated to achieve ejaculation. Semen was collected into a plastic container and immediately frozen with liquid nitrogen. All the samples were shipped to a laboratory in less than 2 h with ice immediately after collection. Samples collections were performed from 2010 to 2019 and stored at −80 °C until analysis.

### 2.2. ^1^H-NMR Analysis

Following Zhu et al. [23], an NMR analysis solution was created. It contained a 10 mmol/L NMR spectra chemical-shift reference in D_2_O, namely, 3-(trimethylsilyl)-propionic-2,2,3,3-d4 acid sodium salt (TSP), set at pH 7.00 ± 0.02 through 1 mmol/L phosphate buffer. Besides, 10 μL of 2 mmol/L NaN_3_ was added to the above liquid in order to avoid microbial proliferation.

Immediately before ^1^H-NMR analysis, seminal plasma samples were thawed and centrifuged for 15 min at 18,630 *g* at 4 °C. Finally, 300 μL of supernatant was mixed with 300 μL of bi-distilled water and 100 μL of NMR analysis solution for ^1^H-NMR analysis. 

The ^1^H-NMR spectra were obtained by means of an AVANCE III spectrometer (Bruker, Chengdu, China) operating at a frequency of 800.30 MHz at 298 K and equipped with the software Topspin 3.5. Following Zhu et al. [24], the signals from broad resonances originating from macromolecules were suppressed by a CPMG filter composed of 400 echoes with a τ of 400 μs and a 180° pulse of 30.48 μs, for a total filter of 332 ms. The HOD residual signal was suppressed by means of presaturation. This was performed by employing the cpmgpr1d sequence, part of the standard pulse sequence library. Each spectrum was acquired by summing 64 transients using 64 K data points over a 16,025.64 Hz spectral window with an acquisition time of 2.025 s.

The spectra phase was manually adjusted in Topspin, while the subsequent adjustments were performed in R computational language by means of a script that was developed in-house [25]. After the residual water signal removal, the ^1^H-NMR spectra were baseline-corrected through peak detection, in accordance with the “rolling ball” principle [26] implemented in the R package named baseline [27]. The chemical shift and multiplicity of signals allowed the molecules’ annotation by taking advantage of the Chenomx software library (ver. 8.3, Chenomx Inc., Edmonton, AB, Canada).

In order to remove the potential confounding factor of dilution [28], the ratio between the area of TSP signal and the total spectrum intensity of each sample was calculated. This allowed us to estimate the median unspecific change in metabolites caused by water and to select the sample characterized by the mostly representative dilution. The molecules were quantified in this sample by employing the added TSP as an internal standard, while any other sample was then normalized by means of probabilistic quotient normalization [28], which was set up to exclude spectra portions ascribed to water and glycerol. The integration of the signals was performed for each molecule by means of rectangular integration.

### 2.3. Statistical Analysis

The statistical analysis was conducted in R computational language and online data analysis platform MetaboAnalyst 5.0 (https://www.metaboanalyst.ca, accessed on 1 March 2022). Prior to the univariate analyses, the distribution of the concentrations of the molecules was brought to normality according to Box and Cox [29]. To demonstrate perturbations caused to single molecules by the effects considered, *t*-tests were applied with a significance limit of *p* < 0.05. To demonstrate perturbations encompassing the metabolome’s profile in its entirety, robust principal component analysis (rPCA) models [30] and heatmaps were employed. The outcome of each rPCA model was constituted by a scoreplot, the projection of the samples in the PC space. This allowed us to highlight the underlying structure of the data. Besides, we calculated Pearson’s correlation plot, relating the concentration of each variable to its importance over each component of the model.

## 3. Results

### 3.1. Characterization of Samples

Samples from 15 healthy captive giant pandas were obtained (Table 1). According to Zhu et al. and Wei et al. [31,32], all the giant pandas were considered to be divided into three groups according to mating age. On average, wild giant pandas come to estrus at 7.5 years and mating age starts at 8.5 years, while captive giant pandas come to estrus earlier than wild ones. In addition, captive giant pandas are considered to be adults when their age is between 5.5 and 20. Therefore, adult phase I comprised animals younger than 8 years of age, adult phase II comprised those between 8 and 12 years of age, and adult phase III comprised animals older than 13. The estrus of the giant panda begins from March to May, so the samples collected in February and March were considered pre-estrus (PRE), those collected in April were considered middle-estrus (MID), and those collected in May and June were considered post-estrus (POS). Focusing on cryogenic storage time, all the samples were considered to pertain to the long storage group when stored for more than 6 years, while they were considered as pertaining to the short storage group otherwise.

### 3.2. Characterization of Semen Plasma Metabolome of Giant Panda

**Figure 1:**
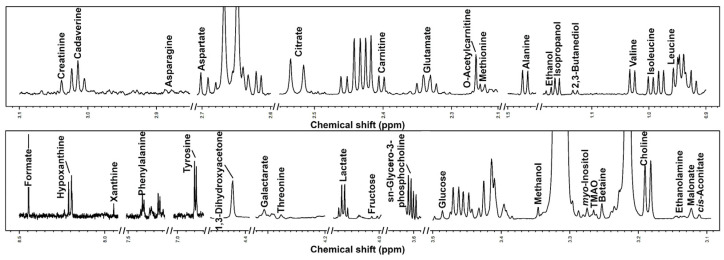
^1^H-NMR spectrum of giant panda seminal plasma, representative of those registered in the present work. The name of each molecule appears over the signal used for its quantification. To ease the reader’s visual inspection, for each portion, a spectrum with a convenient signal-to-noise ratio has been selected.

In giant panda seminal plasma samples, we were able to characterize 35 metabolites. The details of the characterization by ^1^H-NMR are visually represented in Figure 1 through a typical ^1^H-NMR spectrum of the collected samples. The complete list of molecules and their concentrations is reported in the Table 2.

**Figure 2:**
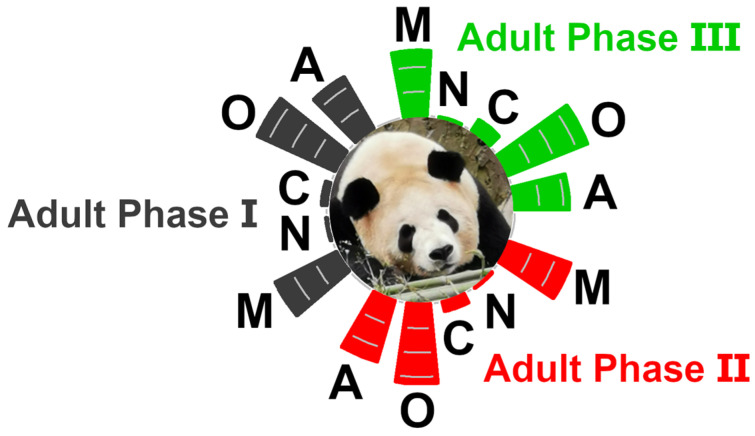
Relative abundances of the classes of molecules assigned in the giant panda seminal plasma metabolome. As a reference, the lines inside the bars highlight 10% steps. C = carbohydrates and derivatives, O = organic acids and derivatives, A = amino acids, peptides, and derivatives, N = nucleosides, nucleotides, and analogues, M = miscellaneous, as suggested in the reports by Zhu et al. [23].

The characterized molecules mainly pertained to the classes of carbohydrates and derivatives, organic acids and derivatives, amino acids, peptides and derivatives, and nucleosides, nucleotides, and analogues. The relative abundances of those classes in the samples from the three panda’s groups can be visually inspected in Figure 2. The overall relative concentrations of amino acids, peptides, and derivatives showed increasing trends with age, specifically, 24.76%, 27.22%, and 29.06% in three groups, respectively. On the contrary, the contents of organic acids and derivatives were negatively related to age, namely, 38.93%, 38.75%, and 36.22%, respectively. Among the quantified molecules, the concentrations of *sn*-glycerol-3-phosphocholine, lactate, citrate, and carnitine were the most represented in each group.

### 3.3. Metabolome’s Age-Related Features 

**Figure 3:**
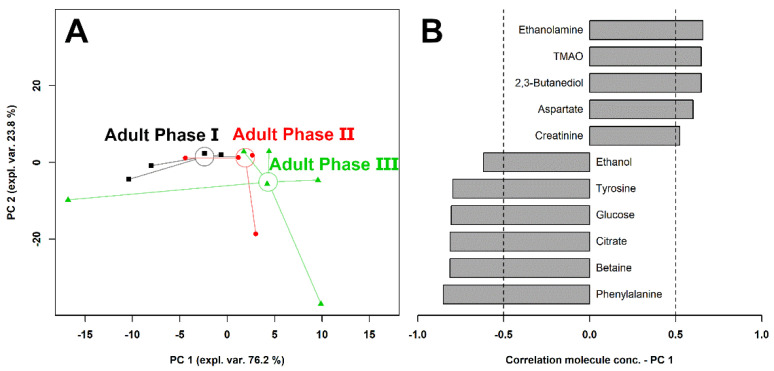
rPCA model built on the metabolomic space constituted by the concentrations of the molecules quantified by ^1^H-NMR. In the scoreplot (**A**), samples from adult phase I, II, and III giant pandas are represented with squares, circles, and triangles, respectively. The wide empty circles represent the medians of the groups. The loading plot (**B**) reports correlations between the concentrations of each metabolite and its importance over PC 1. For readability, only molecules showing significant correlations have been reported.

**Figure 4:**
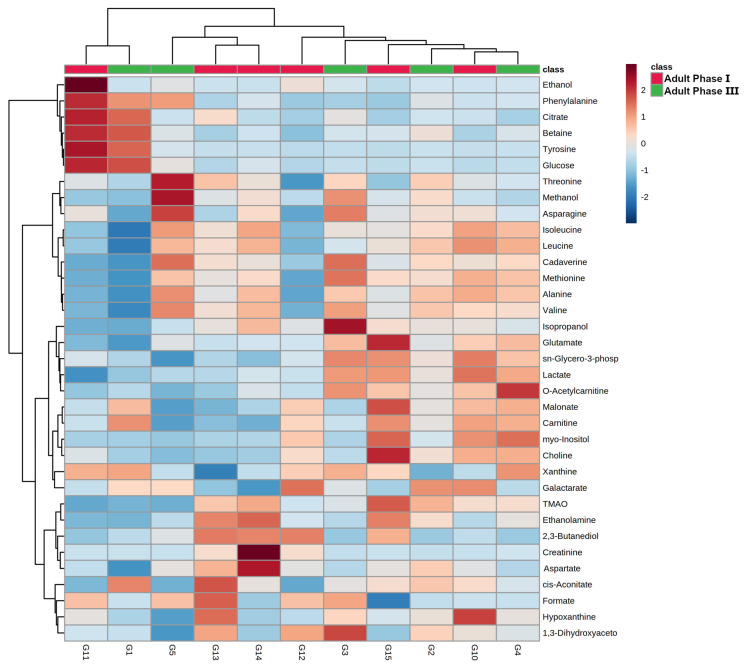
Heatmap built on the metabolomic space constituted by the concentrations of the molecules quantified by ^1^H-NMR.

To evaluate the overall molecular profile trends with age, a robust principal component analysis (rPCA) model (Figure 3) and a heatmap (Figure 4) were set up.

In terms of the rPCA model, the first component represented 76.2% of the variance, summarizing the peculiarities connected to age, with individuals pertaining to adult phase I and III appearing at the low and high PC scores, respectively, and adult phase II individuals at intermediate values. The loading plot of Figure 3B shows that samples from adult phase III were characterized by higher concentrations of ethanolamine, TMAO, 2,3-butanediol, aspartate, and creatinine, while samples from adult phase I showed higher concentrations of ethanol, tyrosine, glucose, citrate, betaine, and phenylalanine (Figure 3B). The common trends of ethanol, tyrosine, glucose, citrate, betaine, and phenylalanine can be best appreciated through a heatmap, as shown in Figure 4. The same applies to the common and opposite trends of ethanolamine, TMAO, 2,3-butanediol, aspartate, and creatinine.

**Figure 5:**
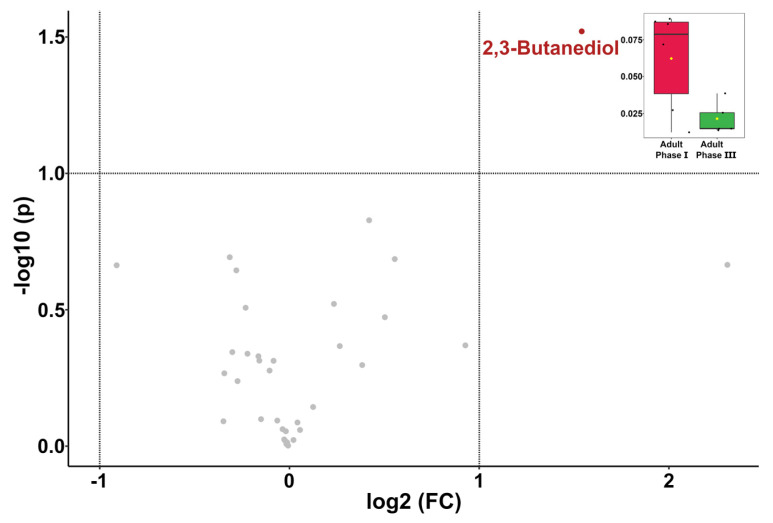
Volcano plot showing the differences in the concentrations of molecules in giant panda seminal plasma from adult phase I and III, studied by *t*-test. The boxplot in the right upper part shows 2,3-butanediol concentrations for each age group.

A comparison between adult phase I and III individuals by a *t*-test highlighted a significant difference in 2,3-butanediol, as shown in the volcano plot in Figure 5.

In addition, we calculated Pearson’s correlation between the age and the concentration of each molecule we quantified. The result showed that a significant positive correlation existed only for 2,3-butanediol (*r* = 0.61 and *p* = 0.02).

### 3.4. Giant Panda Seminal Plasma Metabolome Is Related to the Estrus Phases 

**Figure 6:**
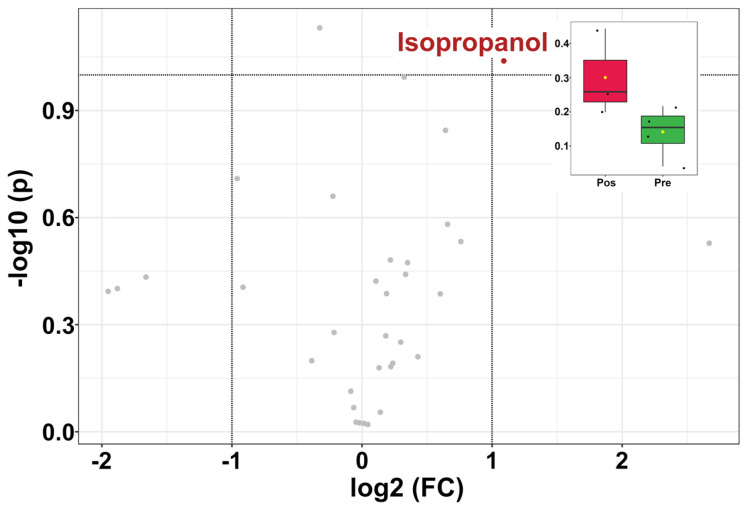
Volcano plot based on the comparisons between the concentrations of molecules in giant panda seminal plasma from the PRE and POST estrus groups. The right upper part shows the comparison for isopropanol.

**Figure 7:**
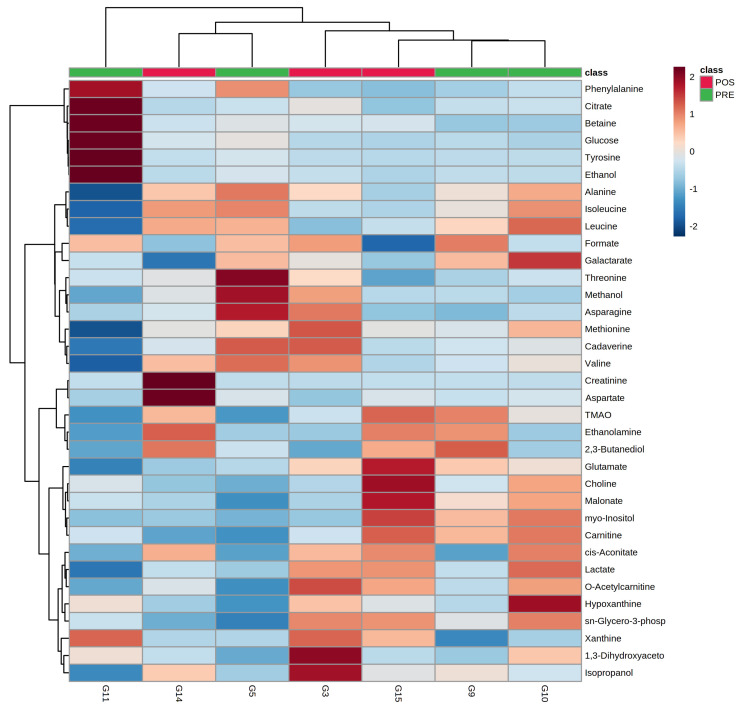
Heatmap built on the metabolomic space constituted by the concentrations of the molecules quantified by ^1^H-NMR.

In parallel with age, a univariate analysis (Figure 6) was set up to study the overall metabolome’s trends connected to estrus. A heatmap was also set up (Figure 7), to observe the molecules’ trend similarities.

As shown in Figure 6, one molecule, namely isopropanol, exhibited significantly higher levels post-estrus than pre-estrus. The heatmap allows us to appreciate that a list of molecules exhibited higher concentrations in the pre-estrus group, such as formate and galactarate. Other molecules, such as isopropanol and 1,3-dihydroxyacetone, were more concentrated in the post-estrus group.

### 3.5. Giant Panda Seminal Plasma Metabolome Is Affected by Storage Time

In the current study, two molecules showed significant differences between the samples that underwent short and a long storage, namely, galactarate (long-storage: 4.72 × 10^−1^ ± 5.86 × 10^−2^ mmol/L, short-storage: 3.89 × 10^−1^ ± 5.03 × 10^−2^ mmol/L, *p* = 0.01) and ethanolamine (long-storage: 1.66 × 10^−1^ ± 6.33 × 10^−2^ mmol/L, short-storage: 2.69 × 10^−1^ ± 9.60 × 10^−2^ mmol/L, *p* = 0.04).

## 4. Discussion

Seminal plasma is a complex biological fluid that is mainly characterized by inorganic ions, low-molecular-weight organic compounds, peptides, and hormones [33]. This organic and inorganic complex mixture is secreted from different endocrine glands and is the functional reflective index of the corresponding glands [34]. In addition, the metabolites present in seminal plasma play several roles related to sperm function, such as energy production, motility, protection, pH control, and the regulation of metabolic activity [35]. To the best of our knowledge, this is the first attempt to acquire metabolomic data from giant panda seminal plasma. A total of 35 molecules were unequivocally quantified, a number much higher than previously obtained in similar studies on other animals [36] or humans [37]. With the kind help of the China Conservation and Research Center for the Giant Panda, we had the opportunity to investigate the effects of age and estrus on giant panda seminal plasma. Age has been widely regarded to be a key factor for semen or/and seminal plasma perturbations. For instance, Chen et al. found, in humans, that semen volume, spermatozoa concentration, total spermatozoa count, motility, total motile sperm, and morphology significantly decreased with age [38]. In the current study, an rPCA model based on the entire set of molecules quantified highlighted distinct trends connected to age. A comparison between age adult phase I and III and Pearson’s correlation showed a specific effect of age on the concentration of 2,3-butanediol. The presence of this molecule in mammalian seminal fluid was recently confirmed by some of the authors of the present work [39] while studying horse’s seminal plasma. It is interesting to notice that 2,3-butanediol is a catabolite of pyruvate, which could not be quantified in the present work due to the superimposition of its signal with some from glutamate. [40] Pyruvate is considered to be one of the major sources of energy for spermatozoa [41]. Higher concentrations of 2,3-butanediol in older individuals could therefore indicate a lower concentration of pyruvate, with detrimental effects on sperm quality. On the other hand, in light of the cryogenic storage practice of semen, it is worth noticing the potential protective role of this molecule, as shown by Jeyendran et al. on bovine spermatozoa [39]. 

Among the molecules showing trends connected to an animal’s age or estrus phase, aspartate has been demonstrated, with particular reference to its D enantiomer, to have an impact on a number of key parameters determining sperm quality [42,43,44]. ^1^H-NMR with water as a solvent cannot separately quantify the D and L enantiomers. However, Macchia et al. noticed that administering rabbits with increasing doses of DL-aspartate caused an increase in the D-aspartate concentration, while the concentration of L-aspartate did not vary [45]. It is therefore tempting to speculate that the higher concentration of aspartate we found in younger individuals witnessed a higher concentration of its D enantiomer with its consequent positive effects, which include increased sperm motility and concentration.

One of the main sugars in the seminal plasma is glucose, which is essential for ATP production and the motility of the spermatozoa [46]. No significant correlation has been found between glucose concentration and fertility in bulls [47]. This finding was confirmed by Cevik et al., even though a significantly higher level of glucose was found in normozoospermic bulls than in oligoasthenozoospermic bulls [48]. Gupta et al. revealed that the concentration of phenylalanine in the seminal plasma of humans can be used as a biomarker to determine infertility [33]. Engel et al. found that the concentrations of phenylalanine in seminal plasma were positively correlated with the volume of the ejaculate, reaching statistical significance. In addition, high creatinine levels were significantly positively correlated with the amount of immotile sperm [49]. This finding agrees with an NMR-based study that showed increased creatinine levels in the seminal plasma of patients with asthenozoospermia [37]. Phenylalanine is an essential aromatic amino acid. Once in the body, most of the phenylalanine is usually converted to tyrosine, which in turn is degraded to levodopa and then to dopamine [50]. Creatinine, a degradation product of creatine, is one of the most abundant biogenic amines in blood serum [23] and is cleared via the kidneys [51]. In the present context, it is worth mentioning that there are reports of elevated creatine kinase activity in subfertile patients [52,53]. Citrate is the main anion of seminal plasma, where it chelates calcium ions and limits sperm capacitation and spontaneous acrosome reactions [54]. Citrate and acid phosphatase can be considered to be markers for prostate activity [55]. Kumar et al. found that high-fertility bulls exhibited low levels of citrate [56]. Such findings are consistent with similar studies in infertile men [57].

The effects of storage time on the metabolome of biomaterials (i.e., urine and feces) have been evaluated in a limited number of papers [58,59], none of which focused on potential changes in giant panda seminal plasma with time. At present, both fresh and thawed frozen giant panda semen samples are successfully employed for AI. A real-life example is represented by the giant panda Mei Xiang, who was inseminated with frozen semen collected 14 years before and recently gave birth to Bao Bao (https://nationalzoo.si.edu/animals/news/how-artificial-insemination-helps-boost-panda populations, accessed on 15 March 2022). Our preliminary data indicate that there are indeed metabolomic changes connected to storage. Whether these modifications would affect the success rate of AI still needs further investigations.

The rarity of giant pandas has limited the availability of samples for the present investigation. To adapt to this limitation, among the various multivariate methods described to highlight the main trends underlying the data, we selected rPCA, an unsupervised method that is not prone to overfitting. This method allowed us to exclude the presence of potential outliers. 

## 5. Conclusions

To the best of our knowledge, this is the first attempt to acquire metabolomic data of giant panda seminal plasma by means of ^1^H-NMR. We were able to characterize 35 molecules in giant panda seminal plasma and to illustrate, with the limits represented by a low number of samples, the effects of age, estrus stages, and storage time on the seminal plasma metabolome, with particular reference to 2,3-butandiol, isopropanol, galactarate, and ethanolamine. For some of them, such as 2,3-butandiol and aspartate, we were able to suggest direct links with sperm quality, thus offering possibilities for novel approaches to giant panda artificial insemination.

## Figures and Tables

**Table 1:** Giant panda seminal plasma samples information.

Sample ID	Age (Years)	Sample Collection Date
G1	6	April 2010
G2	6	April 2013
G3	6	May 2019
G4	6	April 2015
G5	7	March 2016
G6	8	April 2013
G7	12	April 2016
G8	10	April 2019
G9	9	March 2019
G10	13	February 2012
G11	13	February 2012
G12	18	April 2010
G13	13	April 2018
G14	14	June 2018
G15	14	June 2018

**Table 2:** Molecule concentrations (mmol/L, means ± standard deviations) in different groups of giant panda seminal plasma.

Amino Acids, Peptides, and Derivatives	Adult Phase I	Adult Phase II	Adult Phase III
Alanine	8.17 × 10^−1^ ± 2.07 × 10^−1^	9.41 × 10^−1^ ± 7.01 × 10^−2^	7.29 × 10^−1^ ± 1.77 × 10^−1^
Asparagine	6.63 × 10^−1^ ± 1.99 × 10^−1^	5.95 × 10^−1^ ± 8.45 × 10^−2^	5.65 × 10^−1^ ± 9.66 × 10^−2^
Aspartate	9.67 × 10^−1^ ± 2.89 × 10^−1^	1.10 ± 3.77 × 10^−1^	1.29 ± 3.77 × 10^−1^
Carnitine	13.1 ± 5.82	12.9 ± 5.36	12.8 ± 5.83
Creatinine	1.04 × 10^−1^ ± 2.75 × 10^−2^	3.26 × 10^−1^ ± 4.27 × 10^−1^	5.16 × 10^−1^ ± 6.86 × 10^−1^
Glutamate	4.37 ± 8.04 × 10^−1^	5.23 ± 2.54 × 10^−1^	4.50 ± 9.85 × 10^−1^
Isoleucine	5.34 × 10^−1^ ± 2.15 × 10^−1^	6.19 × 10^−1^ ± 2.21 × 10^−2^	5.29 × 10^−1^ ± 1.64 × 10^−1^
Leucine	9.56 × 10^−1^ ± 4.05 × 10^−1^	1.22 ± 1.13 × 10^−1^	9.71 × 10^−1^ ± 3.15 × 10^−1^
Methionine	4.41 × 10^−1^ ± 1.17 × 10^−1^	4.35 × 10^−1^ ± 2.53 × 10^−2^	3.95 × 10^−1^ ± 9.33 × 10^−2^
O-Acetylcarnitine	1.41 ± 7.18 × 10^−1^	9.65 × 10^−1^ ± 1.55 × 10^−1^	1.14 ± 3.82 × 10^−1^
Phenylalanine	7.62 × 10^−1^ ± 3.12 × 10^−1^	6.08 × 10^−1^ ± 1.45 × 10^−1^	6.32 × 10^−1^ ± 4.21 × 10^−1^
Threonine	9.37 × 10^−1^ ± 2.63 × 10^−1^	8.84 × 10^−1^ ± 2.26 × 10^−1^	7.53 × 10^−1^ ± 1.81 × 10^−1^
Tyrosine	2.17 ± 2.57	9.62 × 10^−1^ ± 1.88 × 10^−1^	2.16 ± 3.35
Valine	9.09 × 10^−1^ ± 3.31 × 10^−1^	9.87 × 10^−1^ ± 9.85 × 10^−2^	7.80 × 10^−1^ ± 2.22 × 10^−1^
**Organic acids and derivatives**			
*cis*-Aconitate	3.76 × 10^−1^ ± 8.03 × 10^−2^	3.52 × 10^−1^ ± 1.30 × 10^−1^	3.67 × 10^−1^ ± 9.55 × 10^−2^
Citrate	17.0 ± 11.7	11.3 ± 3.89	16.9 ± 15.1
Formate	8.74 × 10^−1^ ± 8.09 × 10^−2^	9.67 × 10^−1^ ± 1.02 × 10^−1^	8.63 × 10^−1^ ± 1.50 × 10^−1^
Galactarate	4.45 × 10^−1^ ± 5.01 × 10^−2^	4.28 × 10^−1^ ± 4.33 × 10^−2^	4.14 × 10^−1^ ± 9.55 × 10^−2^
Lactate	24.8 ± 6.00	25.8 ± 5.45	23.7 ± 7.77
Malonate	7.43 × 10^−1^ ± 2.79 × 10^−1^	6.93 × 10^−1^ ± 2.46 × 10^−1^	8.11 × 10^−1^ ± 3.13 × 10^−1^
**Carbohydrates**			
Glucose	6.95 ± 9.30	2.39 ± 2.90 × 10^−1^	5.47 ± 10.5
**Nucleosides, nucleotides, and analogs**			
Hypoxanthine	3.53 × 10^−1^ ± 7.11 × 10^−2^	3.42 × 10^−1^ ± 1.23 × 10^−1^	4.15 × 10^−1^ ± 1.08 × 10^−1^
Xanthine	1.33 × 10^−1^ ± 1.75 × 10^−2^	1.47 × 10^−1^ ± 4.38 × 10^−2^	1.26 × 10^−1^ ± 1.67 × 10^−2^
**Miscellaneous**			
1,3-Dihydroxyacetone	8.21 × 10^−1^ ± 4.47 × 10^−1^	6.49 × 10^−1^ ± 2.55 × 10^−1^	8.08 × 10^−1^ ± 2.97 × 10^−1^
2,3-Butanediol	2.14 × 10^−2^ ± 1.08 × 10^−2^	4.10 × 10^−2^ ± 3.42 × 10^−2^	6.22 × 10^−2^ ± 3.39 × 10^−2^
Betaine	3.69 × 10^−1^ ± 1.66 × 10^−1^	2.49 × 10^−1^ ± 6.09 × 10^−2^	2.91 × 10^−1^ ± 2.28 × 10^−1^
Cadaverine	5.10 × 10^−1^ ± 1.51 × 10^−1^	5.23 × 10^−1^ ± 4.58 × 10^−2^	4.21 × 10^−1^ ± 7.30 × 10^−2^
Choline	5.13 × 10^−1^ ± 1.61 × 10^−1^	5.78 × 10^−1^ ± 1.42 × 10^−1^	6.16 × 10^−1^ ± 2.36 × 10^−1^
Ethanol	2.92 × 10^−1^ ± 5.09 × 10^−2^	2.85 × 10^−1^ ± 5.15 × 10^−2^	5.56 × 10^−1^ ± 7.00 × 10^−1^
Ethanolamine	1.56 × 10^−1^ ± 5.11 × 10^−2^	2.89 × 10^−1^ ± 7.71 × 10^−2^	2.29 × 10^−1^ ± 1.10 × 10^−1^
Isopropanol	1.88 × 10^−1^ ± 1.53 × 10^−1^	2.11 × 10^−1^ ± 6.18 × 10^−2^	1.70 × 10^−1^ ± 7.19 × 10^−2^
Methanol	9.94 × 10^−1^ ± 8.35 × 10^−1^	1.23 ± 1.14	5.28 × 10^−1^ ± 2.15 × 10^−1^
myo-Inositol	1.68 ± 1.18	2.34 ± 8.57 × 10^−1^	2.18 ± 1.24
*sn*-Glycero-3-phosphocholine	28.2 ± 11.8	25.3 ± 6.70	29.3 ± 10.4
TMAO	7.27 × 10^−2^ ± 4.52 × 10^−2^	1.46 × 10^−1^ ± 1.22 × 10^−2^	1.03 × 10^−1^ ± 5.23 × 10^−2^

## Data Availability

The data presented in this study are available within the article.
